# Effect of exertion on the biomechanics of walking with load carriage and running in young, healthy women

**DOI:** 10.1038/s41598-026-55814-0

**Published:** 2026-06-05

**Authors:** Jose E. Rubio, Fabricio Canales, Manivannan Subramaniyan, Md. Arifuzzaman Arif, Ankur Padhye, Stacey Meardon, John Willson, Jaques Reifman

**Affiliations:** 1 Department of Defense Biotechnology High Performance Computing Software Applications Institute, Defense Health Agency Research and Development, Medical Research and Development Command, ATTN: FCMR-TT, 504 Scott Street, Fort Detrick, MD 21702-5012 USA; 2 The Henry M. Jackson Foundation for the Advancement of Military Medicine, Inc., Bethesda, MD USA; 3 Department of Physical Therapy, East Carolina University, Greenville, NC USA

**Keywords:** Exertional effects, Individualized models, Load carriage, Musculoskeletal injury, Engineering, Health care, Medical research, Physiology

## Abstract

Musculoskeletal injuries, a major impediment to medical military readiness, are often associated with muscle fatigue caused by prolonged, repeated activities, including running and walking with load carriage. Here, we investigated the biomechanics of women running without a load and walking with load carriage to identify kinetic and kinematic parameters affected by exertion-related changes under both conditions. We collected motion-capture data and computed tomography images from 20 young, healthy women running without a load for 5 km at a subject-specific pace and walking with a 22.7-kg load for 5 km at a speed of 1.5 m/s. We developed individualized musculoskeletal models for each condition and characterized changes in kinetic and kinematic parameters for each subject between the start (0 km) and end of each 5-km session. We found that peak trunk flexion angle increased for both conditions (effect size ≥ 0.80), with the same trend for 18 of the 20 subjects. Peak hip extension, ankle dorsiflexion, and subtalar inversion internal moments had the same directional changes in both conditions, although not always meeting the significance threshold adjusted for multiple comparisons or yielding an effect size ≥ 0.80. In conclusion, this exploratory study identified exertional effects in women running without a load or walking with load carriage that resulted in biomechanical gait changes in the same direction, which could be used to develop countermeasures to reduce the risk of musculoskeletal injury.

**Supplementary Information:**

The online version contains supplementary material available at 10.1038/s41598-026-55814-0.

## Introduction

Musculoskeletal injuries are common during basic combat training (BCT), with 25% of male and 50% of female recruits sustaining at least one injury during this 10-week military entrance training^1^. These injuries can also result in costly medical disability discharges, with an estimated cost of $31,000 per discharge of each recruit during fiscal year 2005^2^. Musculoskeletal injuries are also prevalent in U.S. active-duty personnel, accounting for approximately 2.4 million medical visits and $548 million in patient-care costs during fiscal year 2008^3^. By and large, these musculoskeletal injuries are associated with overuse caused by repeated loading of the muscles and bones during recurring exercise activities, including running and marching with load carriage, for prolonged periods of time^4,5^. Marching with load carriage is a common military activity that increases loading of the joints^6^ and increases injury risk compared to marching without load carriage^7^. Running is another common military training activity that increases loading of the joints compared to walking at similar speeds^8^ and is associated with a high injury risk^9^. Ultimately, physical exertion due to running and walking with load carriage for prolonged periods of time leads to biomechanical compensations by the body^10^ and, potentially, musculoskeletal injury^11^.

Various studies have analyzed changes in the biomechanical responses of the body caused by muscle fatigue induced by running activities, however, the findings are often contradictory. For example, Clansey et al. reported an increase in ankle plantarflexion during initial contact with the ground when healthy men (average age, 36.2 years) ran for 40 min on a level treadmill at their lactate threshold speed^12^. In contrast, Jewell et al. observed an opposite trend when healthy men and women (average age, 26.5 years) ran for 20 min on a level treadmill at their training pace^13^. Due to conflicting evidence between studies, reports based on meta-analyses offer a more holistic interpretation of such disparate results. For example, in a recent meta-analysis of 68 studies investigating running-induced fatigue (54% on a level treadmill), where each study included at least 10 subjects (either men or women), Apte et al. found that fatigue leads to an increase in trunk flexion and knee flexion at initial contact with the ground^10^. In another meta-analysis of 33 studies investigating fatigue induced by running at least 3 km, where each study included healthy men and women between 18 and 65 years old, Zandbergen et al. found that peak knee flexion angle increases in all the studies that analyzed this variable^14^. Whereas many studies have analyzed kinematic parameters, such as joint angles and segment accelerations, most have not considered kinetic parameters, such as joint moments and joint work, limiting our understanding of muscle fatigue induced by running.

With regard to walking with load carriage, only a few single studies (no meta-analyses) have reported the effects of muscle fatigue on joint kinematic parameters. For example, Simpson et al. showed that knee flexion and ankle plantarflexion angles at initial contact with the ground increase due to fatigue when women walked with a load of 20–30% of their body weight for 8 km^15^. In another study, Lidstone et al. reported an increase in trunk flexion induced by fatigue when women walked with a load of 55% of their body weight for 1 h^16^. In contrast, Mullins et al. showed no significant changes in knee, hip, or ankle joint kinematics when men walked with a load of 22.0 kg for 2 h^17^. Except for ground reaction force (GRF), these studies did not analyze any other kinetic parameters.

Previous studies investigated the effects of exertion and muscle fatigue on gait biomechanics across different activities, however, their findings are inconsistent^10,12–17^. Aside from differences in the type of activity investigated, such as running or marching with load carriage, the primary reason for this inconsistency lies in the large between-subject variability due to differences in fitness level^18^, anthropometric measurements^19^, muscle strength^20^, and tendon insertions^21^. Hence, reducing or eliminating between-subject variability by studying the same subject under different physical activities would allow for a more effective assessment of consistent changes in the body’s biomechanical responses to muscle fatigue and provide new insights not previously possible.

The goal of this exploratory study is to investigate whether there are biomechanical gait parameters that show similar directional changes in response to two physical exertion conditions, walking with load carriage and running without load carriage. The rationale for investigating these changes across two distinct physical activities, rather than focusing solely on activity-specific parameters, was not to inform the development of a “one-size-fits-all” training intervention. Instead, our goal was to identify sensitive indicators of exertion or fatigue that are consistent across physical activities. To minimize the effect of between-subject variability, we studied these two conditions using the same cohort of subjects. In particular, we developed individualized musculoskeletal models using experimental data from 20 young, healthy women and investigated changes in kinematic and kinetic biomechanical parameters after they ran for 5 km or walked with a 22.7-kg (50-lb) load for 5 km, as compared to the start of the exercise activity (at 0 km). We hypothesized that trunk flexion angle is a parameter that increases significantly due to physical exertion under both conditions and that kinetic parameters, not frequently reported in the open literature, also change significantly as the body compensates for these physical activities. The knowledge gained in this study could support the development of countermeasures to reduce the risk of musculoskeletal injury by identifying biomechanical parameters that can potentially serve as metrics to monitor the exertion or fatigue state of an individual, thereby informing the implementation of personalized training programs or timely interventions to delay exertion-induced fatigue.

## Methods

### Image acquisition and motion-capture data collection

**Fig. 1:**
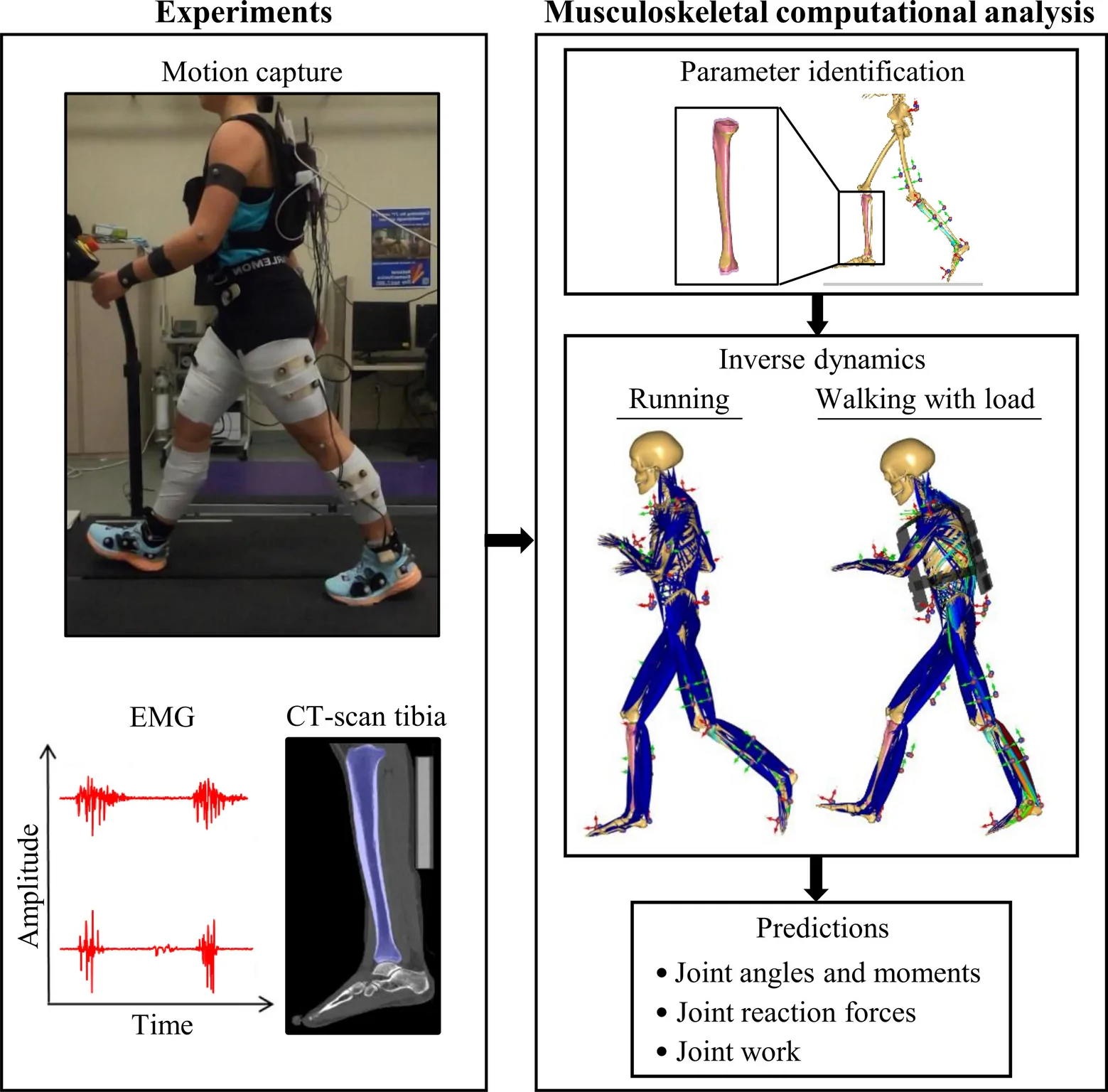
Summary of the integrated experimental and computational study design. CT: Computed tomography; EMG: electromyography.

We enrolled 20 young, healthy women between the ages of 18 and 25 years, who were demographically representative of female military recruits in agreement with an anthropometric survey of U.S. Army Soldiers^22^. We excluded subjects with known health problems, such as cardiovascular or musculoskeletal disorders, and only included subjects who had a documented history of running a minimum of 8 km (5 miles) per week at a pace faster than or equal to 7.5 min/km (12 min/mile). The Institutional Review Board at the East Carolina University and the Office of Human Research Oversight at Fort Detrick, MD, approved the study protocol, and all methods were performed in accordance with the relevant guidelines and regulations. After each subject signed an informed consent, we recorded their age, mass, height, body fat percentage, and body mass index (Table 1) and collected computed tomography (CT) axial plane scans of their entire left tibia using a GE LightSpeed CT Scanner (GE HealthCare, Chicago, IL, USA) (Fig. 1). The scans had a maximum slice thickness and increment of 0.63 mm, with an in-plane resolution of 0.50 × 0.50 mm^2^.

**Table 1:** Anthropometric characteristics of 20 young, healthy women.

	Age (years)	Mass (kg)	Height (m)	Body fat (%)	BMI (kg/m^2^)
Mean (± SD)	22.2 (2.1)	58.3 (7.6)	1.65 (0.08)	25.9 (3.7)	21.4 (2.0)
Range	18–25	41.0–68.3	1.50–1.81	17.8–31.8	18.3–25.5

BMI: Body mass index; SD: standard deviation.

We collected 10 s of data at the beginning of the session (0 km) and at each 1-km mark for each of two conditions: (1) walking at a speed of 1.5 m/s for a total of 5.0 km while carrying a 22.7-kg load and (2) running without a load for a total of 5.0 km at a fixed speed (Fig. 1). For each subject, we estimated the running speed using the Riegel model based on a 3.2-km (2-mile) preliminary trial run time of each subject and an endurance coefficient of 1.06^23^. Specifically, we used the equation ${\mathrm{T}}_{\text{5 km}}={\mathrm{T}}_{\text{3.2 km}}{\left({\mathrm{D}}_{\text{5 km}}/{\mathrm{D}}_{\text{3.2 km}}\right)}^{1.06}$, where T_5 km_ represents the time in minutes required to run 5.0 km, T_3.2 km_ represents the time in minutes required to run 3.2 km, D_5 km_ equals 5000 m, and D_3.2 km_ equals 3200 m. We then reduced the estimated speed by 15% to account for the added instrumentation mass and the laboratory testing environment, and used the reduced value as the treadmill speed during the trials. We randomized the order of the two conditions and required a period of at least 48 h between the two sessions, which was expected to provide sufficient time for neuromuscular function recovery^24^. We collected motion-capture data for each subject as they walked or ran on a split-belt, instrumented treadmill (Bertec FIT; Bertec Corporation, Columbus, OH, USA) using a 12-camera three-dimensional motion-capture system (Qualisys AB, Göteborg, Sweden). We outfitted each subject with laboratory attire and standardized footwear with insoles inside the left and right shoes that recorded plantar pressure (Pedar; Novel Electronics, Inc., Pittsburgh, PA, USA). We placed surface electromyography (EMG) preamplifier electrodes (Motion Lab Systems, Lake Elsinore, CA, USA) over the gluteus medius, rectus femoris, vastus lateralis, tibialis anterior, medial gastrocnemius, and soleus muscles of the left leg according to the Surface Electromyography for the Non-Invasive Assessment of Muscles project (SENIAM) sensor-placement recommendations^25^ and secured them using elastic wraps. We also placed 52 reflective spherical markers on the subject’s upper extremities, trunk, and pelvis as well as bilaterally on the thighs, lower legs, and feet to establish segment coordinate systems and track movement during the exertion protocol (Fig. 1). For both conditions, we measured stride length and collected time-synchronized GRF (2000 Hz), motion-capture (200 Hz), plantar pressure (100 Hz), and EMG (2000 Hz) data. In addition, we measured the heart rate throughout each session and collected the rating of perceived exertion (RPE) at each kilometer^26^.

### Individualized musculoskeletal models and stride analysis

For each subject, under each of the two conditions, we developed a separate computational musculoskeletal model to predict joint kinematics, joint kinetics, and muscle forces based on the experimental marker data and the AnyBody Modeling System (AnyBody Technology, Aalborg, Denmark), as previously described^27–30^. Briefly, we started by using AnyBody’s musculoskeletal model of a generic human that contains rigid segments to represent the arms, legs, feet, trunk, and pelvis as well as 169 muscles in the lower extremities. We included a 22.7-kg (50-lb) vest as a rigid body for the condition involving walking with a load, with straps connecting the vest to the body via two points at the clavicle of the generic human model. Then, we individualized the musculoskeletal model to each subject by scaling the generic human model using the anthropometric measurements of the subject and the length of the tibia measured from the CT scan. In addition, we optimized the length of each body segment by minimizing the distance between the experimentally tracked and the modeled marker positions during a static pose. We also estimated the subject’s foot length based on the plantar pressure data collected from the insoles, using the largest region where the plantar pressure was applied.

For each walking session, using the AnyBody Modeling System, we computed joint kinematics (i.e., the angles and range of motion) using the marker data as input to the individualized musculoskeletal model. Then, we performed inverse dynamic analyses to calculate the forces and moments at the body joints. Briefly, starting from the distal body segments (i.e., the feet), the software defined a coordinate system at the proximal end of each segment and used the predicted kinematics and the measured GRF as inputs to the Newton–Euler equations of motion to calculate the forces (reaction and muscle) and moments at each joint center. To calculate the muscle forces, the software used a muscle recruitment optimization routine to determine the set of muscles that balances the external load on the joint at a given timepoint^31^.

We post-processed the output of the musculoskeletal simulations by conducting stride analyses. We identified the frames corresponding to separate strides by setting a threshold value of 30 N for the GRF at the start of a stride, resulting in 10–12 strides for each set of 10 s of data collected at each 1-km mark^27–30^. We also resampled the biomechanical parameters corresponding to a particular stride in order to define 100 values for each gait cycle. We applied this sampling procedure to the output parameters of the model, including the joint angles, joint reaction forces (JRFs), and joint internal moments. We normalized the GRFs and the JRFs by the subject’s body weight and normalized the joint moments by their body mass, because these normalization factors have been shown to significantly reduce the variability introduced by height and weight^32,33^. In addition, we normalized the stride length by the body height. We also analyzed the positive work (energy generated) and negative work (energy absorbed) in the sagittal plane at each joint while only considering the stance phase of the gait cycle, because the energy required in the swing phase is negligible^34^.

### Statistical analysis

To estimate the number of subjects needed for the study, we performed a power analysis using the G*Power software (v3.1.9.4). We based our analysis on the study of Lidstone et al.^16^ who measured changes in biomechanical and physiological parameters over the course of a 1-hour walk at 1.5 m/s while subjects carried a load equivalent to 55% of their body mass (~ 33 kg). In their study, they observed significant changes in the measured parameters, obtaining an effect size with a partial η^2^ of 0.4. We assumed an alpha-level of 0.05, a power (1-beta) of 0.80, a correlation among repeated measures of 0.50, and a non-sphericity correction of 1.00, which resulted in a sample size of at least 12 subjects. We used a conservative sample size of 20 for this study.

**Fig. 2:**
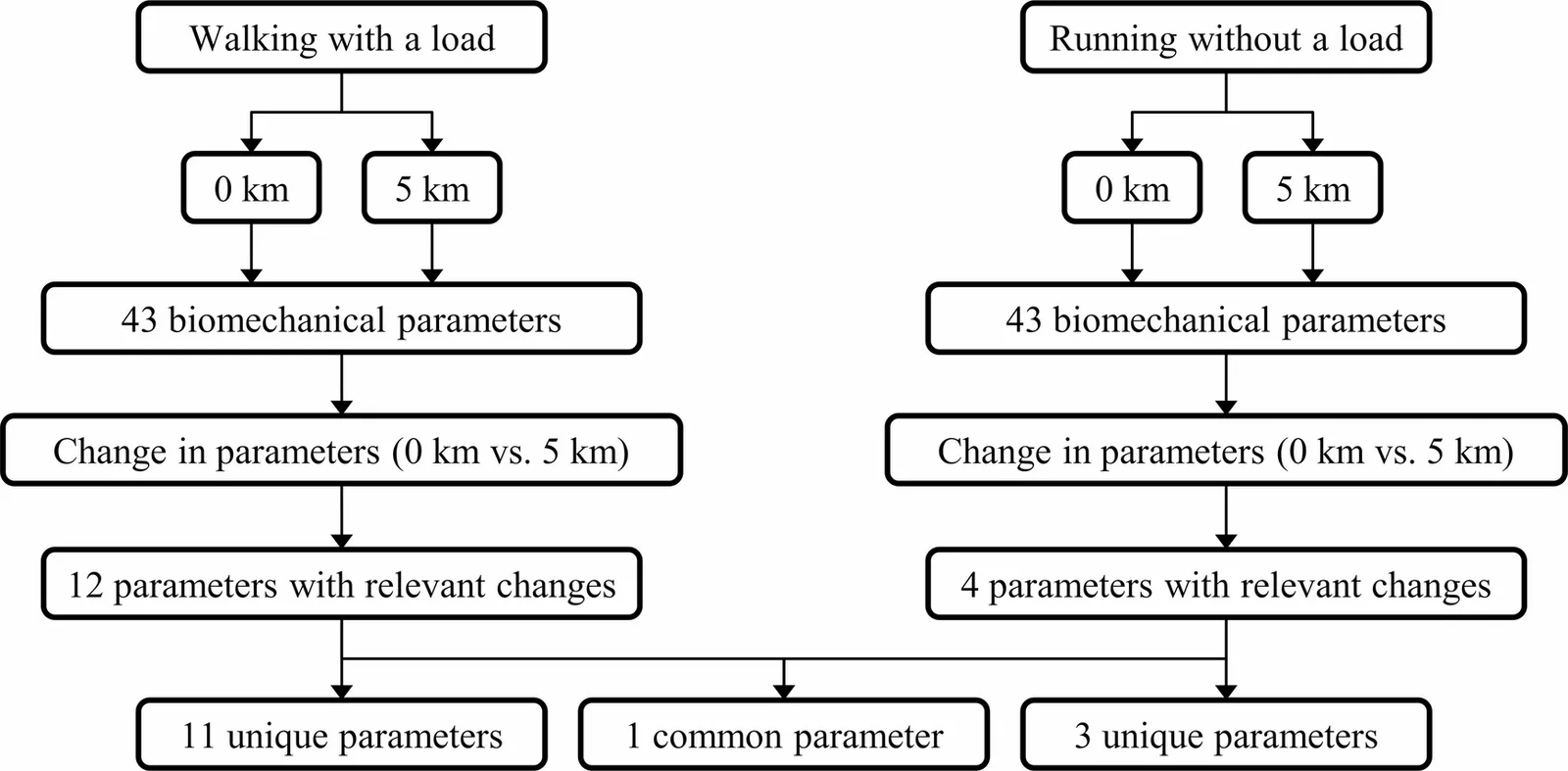
Flowchart for identification of relevant common and unique biomechanical parameter changes at the end of 5 km for the two conditions of the study, walking with a 22.7-kg load and running without a load.

We analyzed a total of 43 biomechanical parameters, including the peak and initial joint angles of the trunk, hip, knee, and ankle, the peak joint moments, the peak JRFs, the stride length, and the GRFs. In addition, we analyzed the energy generation and absorption at each joint separately and as a sum of the values of the hip, knee, and ankle joints. We chose to investigate these parameters because they have been studied before for the case of running without a load and because they are estimated by the biomechanical models. We investigated changes between the values of these parameters measured at the start of the session (at 0 km) and at the end of the session (at 5 km) for both the walking and running conditions (Fig. 2). We used the subject as the inferential unit of analysis. For each subject (*N* = 20), we computed the mean of each of the 43 biomechanical parameters across the 10–12 strides of each set of collected data, which yielded one value per subject and per distance for a given condition. Then, we computed the difference between paired observations (0 km vs. 5 km) for each subject. We used the Shapiro–Wilk test^35^ to determine whether the biomechanical parameters were normally distributed. Because not all parameters were normally distributed, we used the Wilcoxon signed-rank non-parametric test^36^ with Bonferroni correction^37^ to test for statistically significant changes between 0 km vs. 5 km, performing a total of 43 comparisons (one per biomechanical parameter). In addition, we computed the effect size using the rank-biserial correlation *r* to quantify the magnitude of the changes^38,39^. We used the guidelines suggested by Peres^40^ and adapted from Vargha and Delaney^41^ to interpret the value of the rank-biserial correlation effect size: 0.01 ≤ |*r*| < 0.28 (small), 0.28 ≤ |*r*| < 0.43 (medium), and 0.43 ≤ |*r*| ≤ 1.00 (large). In this exploratory study, we defined biomechanical parameters with “relevant changes” as those that met at least one of two criteria: (1) a rank-biserial correlation |*r*| effect size larger than or equal to 0.80, which we used to identify meaningful changes from baseline (0 km) without implying statistical significance, or (2) a Bonferroni-corrected *p*-value smaller than 0.0012 (0.05/43). Based on these study-specific criteria, we then identified parameters that showed relevant changes in the same direction in both conditions, as well as parameters that were relevant only within each condition (Fig. 2). The identification of these biomechanical parameters can inform the development of intervention strategies to delay the onset of fatigue.

## Results

### Model assessment

To assess whether the model outputs were consistent with expected physiological patterns, we qualitatively compared the muscle activities predicted by the model with the EMG recordings collected at the start (at 0 km) of the experimental phase of the study. We did not perform a quantitative comparison because the predicted muscle activity and the measured EMG signals represent different parameters. EMG measures the electrical signal generated by the muscle in response to neural stimulation. In contrast, the computational model predicts the muscle activity as the force produced by the muscle normalized by its maximum strength. Although these parameters are not equivalent, both reflect the level of muscle activation and can be compared qualitatively.

**Fig. 3:**
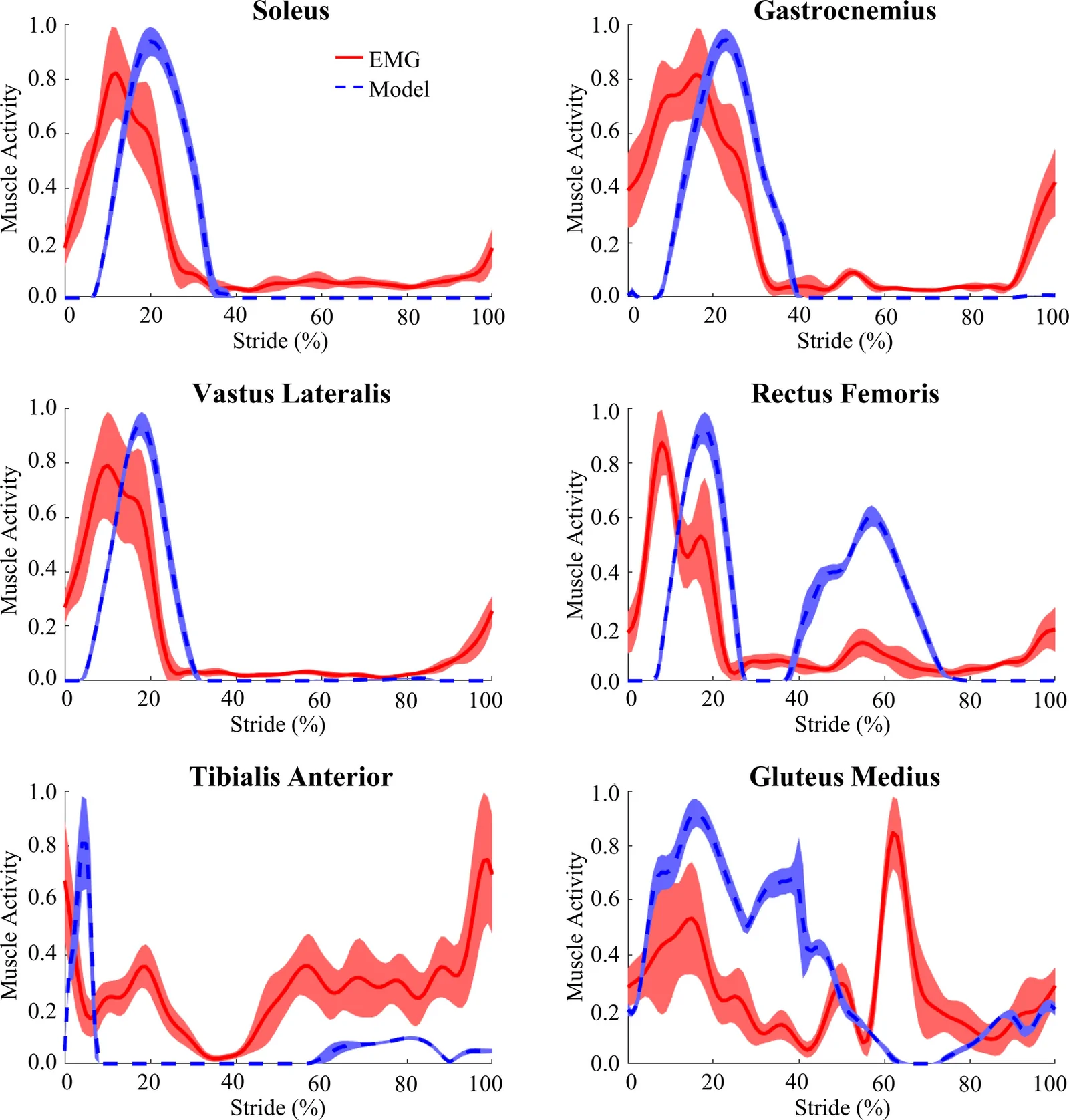
Comparison of muscle activity while running without a load for a representative subject. We compared the results measured via electromyography (EMG) in the experimental study (continuous red line) against the predicted values from the musculoskeletal model (dashed blue line). The shaded areas represent the stride-averaged envelopes (mean ± 1 standard deviation). The values were separately normalized with respect to the largest value among the strides.

**Fig. 4:**
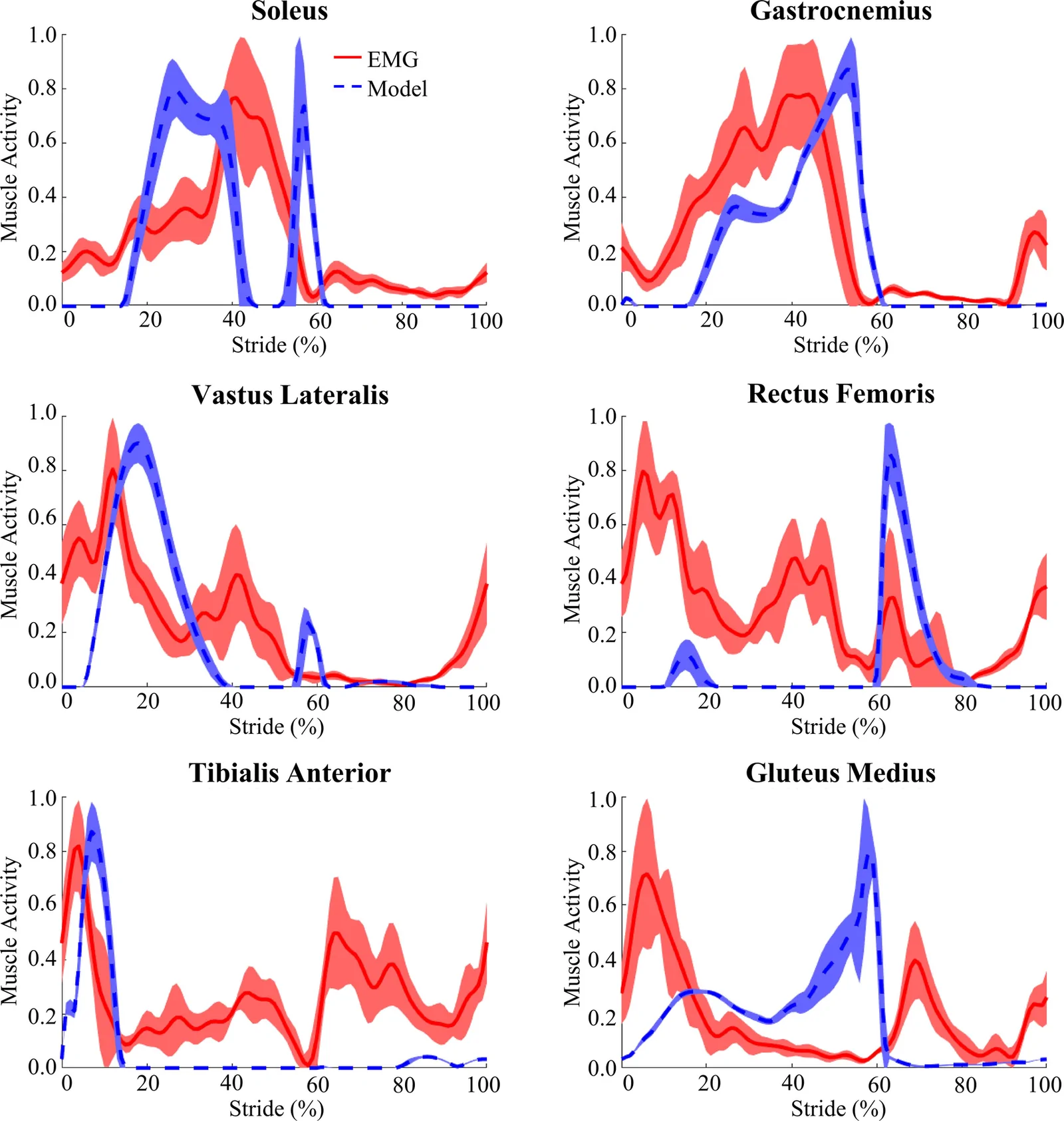
Comparison of muscle activity while walking with a load for a representative subject. We compared the results measured via electromyography (EMG) in the experimental study (continuous red line) against the predicted values from the musculoskeletal model (dashed blue line). The shaded areas represent the stride-averaged envelopes (mean ± 1 standard deviation). The values were separately normalized with respect to the largest value among the strides.

We performed this qualitative comparison for six muscles distributed throughout the body, including the soleus, gastrocnemius, vastus lateralis, rectus femoris, tibialis anterior, and gluteus medius muscles. We normalized both the predicted muscle activities and the EMG recordings by their corresponding maximum value recorded during the 10 s of data collection, and split the data corresponding to separate strides using the 30-N threshold on the GRF. Here, we carried out this comparison for a representative subject, chosen as the subject with height closest to the mean height of all subjects. Figure 3 shows the qualitative comparison between the model-predicted muscle activities (dashed blue lines) and the EMG recordings (continuous red lines) for running without a load, and Fig. 4 shows this comparison for walking with a load. For the case of running without a load (Fig. 3), we observed good qualitative agreement between the model predictions and EMG recordings for the soleus, gastrocnemius, and vastus lateralis muscles after taking into account electromechanical delay^42^, which introduces a time lag between the start of the muscle activation in the experiment (recorded by EMG) and the predicted onset of muscle activity estimated by the model. As expected, for running, both the EMG recordings and the model predictions showed activation of the tibialis anterior during the early-stance phase to absorb the shock of the initial contact with the ground, as well as the activation of the soleus, gastrocnemius, vastus lateralis, and rectus femoris during the mid-stance phase to propel the body forward. For the case of walking with a load (Fig. 4), we observed good qualitative agreement between the model predictions and EMG recordings for the vastus lateralis, gastrocnemius, and the early-stance tibialis anterior muscle recordings after taking into account both the electromechanical delay and the nonlinear relation between EMG and muscle force^43^. For both conditions, we observed large differences between the model predictions and EMG recordings for the gluteus medius muscle, which could be partially attributed to the moment-arm definition of this muscle in the model. In addition, these differences could be explained by considering that the gluteus medius is smaller than the surrounding muscles in the hip region, therefore leading to significant cross-talk between EMG signals^43,44^.

### Changes in biomechanical parameters

After subjects ran for 5 km without a load, on average, the heart rate increased by 26% (Supplementary Table S1). Fourteen out of the 20 subjects reported an increase in RPE from extremely light, very light, or light to at least hard on the Borg scale, with some of these subjects perceiving higher ratings at the 5-km mark (Supplementary Table S1). In addition, for running without a load, we observed four biomechanical parameters with a rank-biserial correlation |*r*| effect size larger than or equal to 0.80 (Tables 2, 3 and 4 and Supplementary Tables S2–S4). After running for 5 km and relative to baseline, the peak ankle dorsiflexion internal moment decreased (Supplementary Table S3), had the largest effect size (ES = − 0.97; Table 3), and met the Bonferroni-adjusted significance threshold (*p* < 0.0012), with an observed decrease in 18 of the 20 subjects. The hip joint negative work in the sagittal plane decreased (Supplementary Table S4), had a large effect size (ES = − 0.86; Table 4), and achieved the Bonferroni-adjusted significance threshold. The peak trunk flexion angle increased (Supplementary Table S2), had a large effect size (ES = 0.80; Table 2), and showed a consistent increase in 18 of the 20 subjects. Although this increase in trunk flexion angle did not meet the strict Bonferroni-corrected significance threshold (*p* = 0.0017), the effect size larger than or equal to 0.80 met the study-specific relevant-change criterion. The initial knee flexion angle increased after running 5 km compared to 0 km in 18 of the 20 subjects with a large effect size (ES = 0.83; Table 2) and met the Bonferroni-corrected significance threshold. The remaining biomechanical parameters had effect sizes smaller than 0.80, with some approaching this threshold but not reaching the significance threshold. For example, the peak hip abduction angle increased in 17 of the 20 subjects (ES = 0.70; *p* = 0.0064; Table 2 and Supplementary Table S2), whereas the peak hip extension internal moment increased in 17 of the 20 subjects (ES = 0.71; *p* = 0.0051; Table 3 and Supplementary Table S3). Finally, the stride length had a decreasing trend after running 5 km compared to 0 km (ES = − 0.50; *p* = 0.0673; Table 2 and Supplementary Table S2).

**Table 2:** Effect size and statistical changes for spatiotemporal and joint kinematic parameters after walking 5 km with a load or running 5 km without a load.

Biomechanical parameter	Walking with a 22.7-kg load			Running without a load		
	Effect size		*p*-value	Effect size		*p*-value
**Normalized stride length**	0.57 (0.12, 0.91)		0.0251	− 0.50 (− 0.87, − 0.01)		0.0522
**Peak joint angle (degree)**						
Hip						
Flexion	0.61 (0.16, 0.90)		0.0169	0.06 (− 0.46, 0.53)		0.8228
Extension	0.51 (0.05, 0.87)		0.0438	0.07 (− 0.43, 0.56)		0.7938
Abduction	0.26 (− 0.26, 0.72)		0.3135	0.70 (0.31, 1.00)		0.0064
Adduction	**0.88 (0.60**,** 1.00)**		**0.0006**	0.75 (0.36, 0.95)		0.0032
External rotation	0.69 (0.32, 0.95)		0.0072	0.68 (0.30, 0.95)		0.0080
Internal rotation	0.55 (0.10, 0.94)		0.0304	− 0.10 (− 0.61, 0.39)		0.6813
Knee						
Flexion	**0.87 (0.60**,** 1.00)**		**0.0007**	0.42 (− 0.10, 0.82)		0.1005
Minimum flexion	− 0.42 (− 0.81, 0.10)		0.1005	0.74 (0.37, 1.00)		0.0036
Ankle						
Plantarflexion	0.47 (0.00, 0.83)		0.0674	− 0.19 (− 0.71, 0.32)		0.4553
Dorsiflexion	0.57 (0.16, 0.90)		0.0251	0.67 (0.28, 0.93)		0.0090
Trunk						

Flexion	**0.94 (0.80**,** 1.00)**		**0.0002**	0.80 (0.47, 0.97)		0.0017
Minimum flexion	**0.90 (0.70**,** 1.00)**		**0.0004**	0.79 (0.46, 0.99)		0.0019
**Initial joint angle (degree)**						
Hip						
Flexion	0.41 (− 0.07, 0.84)		0.1084	− 0.17 (− 0.70, 0.33)		0.5016
Abduction	0.29 (− 0.23, 0.74)		0.2627	– –		–
Adduction	– –		–	0.74 (0.37, 0.98)		0.0036
External rotation	− 0.11 (− 0.63, 0.40)		0.6542	− 0.19 (− 0.65, 0.31)		0.4553
Knee						
Flexion	0.71 (0.33, 0.97)		0.0051	**0.83 (0.54**,** 1.00)**		**0.0011**
Ankle						
Dorsiflexion	− 0.18 (− 0.63, 0.30)		0.4781	− 0.50 (− 0.90, − 0.03)		0.0522
Trunk						
Flexion	**0.97 (0.87**,** 1.00)**		**0.0001**	0.78 (0.45, 0.98)		0.0022

We calculated the effect size using the rank-biserial correlation *r* and the *p*-value using the Wilcoxon signed-rank test based on all 20 subjects. Negative effect size values indicate that the biomechanical parameter decreased after walking or running 5 km compared to 0 km (i.e., the baseline). The values in parentheses show the 95% confidence interval of the effect size. Bold font indicates that the parameter increase or decrease was statistically significant [*p* < 0.0012 (0.05/43) after applying the Bonferroni correction for multiple comparisons] after walking or running 5 km compared to 0 km. The shaded row indicates the parameter that was consistently altered both when walking with a load and running without a load.

**Table 3:** Effect size and statistical changes for ground reaction force, joint internal moment, and joint reaction force after walking 5 km with a load or running 5 km without a load.

Biomechanical parameter	Walking with a 22.7-kg load			Running without a load		
	Effect size		*p*-value	Effect size		*p*-value
**Ground reaction force (BW)**	0.53 (0.05, 0.89)		0.0366	− 0.11 (− 0.62, 0.37)		0.6542
**Peak joint moment (N·m/kg)**						
Hip						
Flexion	− 0.61 (− 0.91, − 0.18)		0.0169	0.37 (− 0.16, 0.86)		0.1454
Extension	**0.91 (0.71**,** 1.00)**		**0.0003**	0.71 (0.34, 1.00)		0.0051
Abduction	0.28 (− 0.21, 0.75)		0.2790	− 0.26 (− 0.72, 0.26)		0.3135
Adduction	**0.92 (0.72**,** 1.00)**		**0.0003**	0.48 (0.01, 0.85)		0.0620
External rotation	0.75 (0.41, 1.00)		0.0032	− 0.07 (− 0.57, 0.43)		0.7938
Internal rotation	− 0.41 (− 0.83, 0.09)		0.1084	− 0.05 (− 0.51, 0.50)		0.8519
Knee						
Flexion	0.82 (0.50, 1.00)		0.0013	0.38 (− 0.11, 0.78)		0.1354
Extension	0.67 (0.24, 0.99)		0.0090	− 0.47 (− 0.86, − 0.01)		0.0674
Ankle						
Plantarflexion	− 0.36 (− 0.76, 0.14)		0.1560	− 0.10 (− 0.60, 0.40)		0.7089
Dorsiflexion	− 0.75 (− 0.99, − 0.38)		0.0032	**− 0.97 (− 1.00**, **− 0.86)**		**0.0001**
Subtalar eversion	− 0.32 (− 0.74, 0.16)		0.2043	0.30 (− 0.18, 0.76)		0.2322
Subtalar inversion	**− 0.97 (− 1.00**, **0.86)**		**0.0001**	− 0.32 (− 0.73, 0.17)		0.2043
**Peak joint reaction force (BW)**						
Hip	0.78 (0.46, 0.97)		0.0022	0.22 (− 0.33, 0.65)		0.3905
Knee	0.54 (0.10, 0.90)		0.0333	0.01 (− 0.47, 0.53)		0.9702
Ankle	− 0.24 (− 0.68, 0.29)		0.3507	0.27 (− 0.25, 0.70)		0.2959

We calculated the effect size using the rank-biserial correlation *r* and the *p*-value using the Wilcoxon signed-rank test based on all 20 subjects. Negative effect size values indicate that the biomechanical parameter decreased after walking or running 5 km compared to 0 km (i.e., the baseline). The values in parentheses show the 95% confidence interval of the effect size. Bold font indicates that the parameter increase or decrease was statistically significant [*p* < 0.0012 (0.05/43) after applying the Bonferroni correction for multiple comparisons] after walking or running 5 km compared to 0 km. BW, body weight.

**Table 4:** Effect size and statistical changes for joint and total positive and negative work after walking 5 km with a load or running 5 km without a load.

Biomechanical parameter	Walking with a 22.7-kg load			Running without a load		
	Effect size		*p*-value	Effect size		*p*-value
**Joint energy generation (J/kg)**						
Hip	0.80 (0.51, 0.98)		0.0017	0.61 (0.16, 0.93)		0.0169
Knee	**0.93 (0.75**,** 1.00)**		**0.0003**	− 0.19 (− 0.65, 0.32)		0.4553
Ankle	− 0.28 (− 0.70, 0.25)		0.2790	− 0.68 (− 0.94, − 0.29)		0.0080
**Joint energy absorption (J/kg)**						
Hip	− 0.09 (− 0.59, 0.39)		0.7369	**− 0.86 (− 1.00**, **− 0.59)**		**0.0008**
Knee	− 0.49 (− 0.88, 0.00)		0.0569	− 0.64 (− 0.92, − 0.21)		0.0124
Ankle	0.23 (− 0.28, 0.69)		0.3703	0.68 (0.24, 0.98)		0.0080
**Total energy generation (J/kg)**	0.82 (0.50, 0.99)		0.0013	− 0.15 (− 0.61, 0.39)		0.5503
**Total energy absorption (J/kg)**	− 0.11 (− 0.61, 0.41)		0.6542	− 0.36 (− 0.77, 0.12)		0.1560

We calculated the effect size using the rank-biserial correlation *r* and the *p*-value using the Wilcoxon signed-rank test based on all 20 subjects. Negative effect size values indicate that the biomechanical parameter decreased after walking or running 5 km compared to 0 km (i.e., the baseline). The values in parentheses show the 95% confidence interval of the effect size. Bold font indicates that the parameter increase or decrease was statistically significant [*p* < 0.0012 (0.05/43) after applying the Bonferroni correction for multiple comparisons] after walking or running 5 km compared to 0 km.

After subjects walked for 5 km with a 22.7-kg load, on average, the heart rate increased by 19% (Supplementary Table S1). Fifteen out of the 20 subjects reported an increase in RPE from extremely light, very light, or light to at least hard on the Borg scale, with some of these subjects perceiving higher ratings at the 5-km mark (Supplementary Table S1). In addition, for walking with a load, we observed that 12 biomechanical parameters met the threshold of a rank-biserial correlation |*r*| effect size larger than or equal to 0.80 (Tables 2, 3 and 4 and Supplementary Tables S2–S4). After walking for 5 km with a load, the peak ankle subtalar inversion internal moment decreased (Supplementary Table S3) with the largest effect size (ES = − 0.97; Table 3) and met the Bonferroni-corrected significance threshold (*p* < 0.0012), with an observed decrease in 18 of the 20 subjects. In terms of the kinematic parameters in Table 2 and Supplementary Table S2, the peak trunk flexion, minimum trunk flexion, initial trunk flexion, peak knee flexion, and peak hip adduction angles increased, had large effect sizes, and achieved the Bonferroni-corrected significance threshold. In terms of the kinetic parameters in Table 3 and Supplementary Table S3, the peak hip extension internal moment and the peak hip adduction internal moment both increased and had an effect size larger than 0.80. They also reached the significance threshold, whereas that of the peak knee flexion internal moment did not (*p* = 0.0013). With regard to the joint energy generation parameters in Table 4 and Supplementary Table S4, the hip, knee, and total joint energy generation increased and had effect sizes larger than or equal to 0.80, but only the knee joint positive work reached the Bonferroni-corrected significance threshold. The other biomechanical parameters had effect sizes smaller than 0.80. In particular, the stride length increased after walking 5 km compared to 0 km (ES = 0.57; *p* = 0.0251; Table 2 and Supplementary Table S2).

When comparing the results for the two conditions, we observed that the peak trunk flexion angle was the only biomechanical parameter that increased with an effect size larger than or equal to 0.80 for both conditions, although it met the Bonferroni-corrected significance threshold (*p* < 0.0012) only for walking with a load. Other parameters had the same trend for the two conditions, but in one condition the rank-biserial correlation |*r*| effect size was greater than 0.80 and in the other condition it was smaller. For example, the peak hip extension internal moment had an increasing trend for both running without a load (ES = 0.71; Table 3 and Supplementary Table S3) and walking with a load (ES = 0.91; Table 3 and Supplementary Table S3), whereas the peak ankle dorsiflexion internal moment had a decreasing trend for both conditions, with a larger effect size for running without a load (ES = − 0.97; Table 3 and Supplementary Table S3) and a relatively smaller effect size for walking with a load (ES = − 0.75; Table 3 and Supplementary Table S3). Similarly, the peak ankle subtalar inversion internal moment had a decreasing trend for both conditions, with a large effect size for walking with a load (ES = − 0.97; Table 3 and Supplementary Table S3) but a medium effect size for running without a load (ES = − 0.32; Table 3 and Supplementary Table S3). Interestingly, we observed that walking with a load yielded a threefold increase in the number of parameters that met the study-specific relevant-change criteria compared with running without a load (Fig. 2). We speculate that this difference may be attributed to inherent differences in gait biomechanics between the two physical activities (Supplementary Tables S1–S3). Accordingly, it is also possible that load carriage represented a greater physiological stressor than running, even when the perceived exertion was rated as “hard” or higher by most subjects in both conditions at the 5-km mark.

## Discussion

In this exploratory study, we aimed to investigate whether physical exertion due to walking with a load or running without a load caused biomechanical parameters to change in the same direction. Towards this goal, we collected laboratory experimental data for 20 adult women, developed musculoskeletal models individualized to each subject for each condition, and computed 43 distinct biomechanical parameters for subjects walking with a load for 5 km and running without a load for 5 km. To reduce between-subject differences, we collected data from the same subjects for the two conditions. We compared the parameter values at baseline (at 0 km) with the values at the end of the session (at 5 km) in order to assess changes that could be associated with exertion. For running without a load, we identified four biomechanical parameters that met the study-specific relevant-change criteria, i.e., those with a rank-biserial correlation |*r*| effect size larger than or equal to 0.80 or those with a *p*-value smaller than the Bonferroni-corrected significance threshold (*p* < 0.0012). The peak ankle dorsiflexion internal moment decreased, had the largest effect size, and met the Bonferroni-corrected significance threshold, whereas the hip joint negative work decreased with a slightly smaller effect size but also reached the significance threshold. For walking with a 22.7-kg load, we identified 12 biomechanical parameters that achieved the study-specific relevant-change criteria. The peak ankle subtalar inversion internal moment decreased and the initial trunk flexion angle increased, had the largest effect sizes, and reached the significance threshold, whereas seven other parameters had a large effect size and also reached the significance threshold.

First, we identified parameters with common trends for both running without a load and walking with a load. Specifically, these parameters either had a rank-biserial correlation |*r*| effect size larger than or equal to 0.80 or a *p*-value smaller than the Bonferroni-corrected significance threshold (*p* < 0.0012), with changes occurring in the same direction across both conditions, without implying similarity in effect magnitude. We observed that the peak trunk flexion angle increased at the 5-km mark for both conditions with a large effect size, which is consistent with previously reported changes observed when running^10^ or walking with a load^16,45^. An increase in trunk flexion angle has been associated with a high hip extension moment^46^. Accordingly, we speculate that the observed increase in trunk flexion represents a compensatory adjustment, helping to move the body forward by shifting the center of mass anteriorly and assisting in load carriage by counteracting the moments around the hip to stabilize body posture during periods of high exertion^47–49^. In addition, an increase in trunk flexion increases the activity of the lower-back muscles, including the erector spinae^50^, as these muscles generate additional forces to stabilize the joints^51^. Consequently, these muscle forces are redistributed from the active musculature to passive structures^52,53^, such as the intervertebral discs, increasing the stresses in these structures^50^. Taken together, these load redistributions triggered by an increase in trunk flexion are clinically relevant for military personnel with an occupational exposure to load carriage^54^, as increases in muscle loading and intervertebral disc stress have been associated with an increase in risk of lower-back pain and disc degeneration^55,56^.

None of the other parameters met the study-specific relevant-change criteria for both conditions. However, we found that the peak hip extension internal moment increased at the 5-km mark compared to baseline for both conditions, with a large effect size and reaching the Bonferroni-corrected significance threshold only for walking with a load, which is consistent with previous findings for the same load-carriage condition^6^. Albeit not meeting the significance threshold, the peak ankle plantarflexion internal moment showed a marginal decreasing trend under both conditions, which is consistent with previous studies reporting minimal reductions in this parameter when running up to a hard RPE^57–59^. These results suggest that the plantarflexor muscles maintain their key role in generating vertical support forces even at hard levels of exertion.

Next, we considered parameters that only achieved the study-specific relevant-change criteria for running without a load in order to identify unique changes for this condition. The predicted decrease in peak ankle dorsiflexion internal moment was the most pronounced change, with the largest effect size. While multiple muscles contribute to the generation of the ankle dorsiflexion moment, we speculate that the reduction in this moment likely reflects fatigue-related changes in dorsiflexor muscle function, as observed in prior studies. For example, based on the mean power frequency of EMG recordings, a previous study has shown that the tibialis anterior activity, i.e., a relatively large dorsiflexor muscle, decreases significantly during extended running due to fatigue^60^. In addition, we analyzed the vertical GRF at initial contact and observed an increasing trend with a medium effect size (ES = 0.38) after running for 5-km, which likely indicates an increase in impact loading at the foot due to exertion. We also observed that the energy absorbed at the hip joint in the sagittal plane decreased at the 5-km mark and met the Bonferroni-corrected significance threshold, which has been associated with a lower metabolic power requirement during running^34^. Thus, we speculate that this could be a gait compensation mechanism used to increase running efficiency as subjects become fatigued. We also found that the peak knee flexion angle at contact with the ground increased at the 5-km mark and met the significance threshold, in agreement with previous studies^10,14,61^.

We identified parameters that did not reach the study-specific relevant-change criteria for running without a load but had directional changes like those identified in prior studies. Although these parameters had effect sizes smaller than 0.80, did not reach the Bonferroni-corrected significance threshold, and should not be construed as supporting evidence, they showed a qualitative agreement with previously reported trends. For example, we observed that the peak hip adduction angle during the mid-stance increased at the 5-km mark. A previous study has reported increased peak hip adduction angles under running-induced fatigue in both male and female runners^62^. In contrast, other studies reported minimal to no change in peak hip adduction angle under exerted states^63–65^. This discrepancy can be attributed to the individual’s training status, given that well-trained individuals often exhibit strong hip adductor stabilizer muscles, which limit increases in the hip adduction angle^12,65^. The peak vertical GRF has been reported to decrease after prolonged running in a recent meta-analysis involving studies investigating running-induced fatigue^10^. In agreement, we observed a decreasing trend for 12 out of 20 subjects for the peak vertical GRF, however, this parameter had a small effect size (ES = − 0.11) and did not meet the Bonferroni-corrected significance threshold. Similarly, we observed a decreasing trend for 13 out of 20 subjects without reaching the significance threshold for the stride length at the 5-km mark. Because in the study subjects ran on a treadmill at a constant speed, they reduced their stride length while increasing their step rate to maintain the speed under exertion. In fact, an increase in step rate has been shown to reduce certain kinematic (e.g., peak knee flexion angle) and kinetic parameters (e.g., peak hip adduction moment) at the lower-body joints^66^, while a decrease in stride length has been shown to reduce stress-fracture risk associated with running activities for a 10-week military training program^67^.

Regarding parameter changes for walking with a load, we found that the reduction in peak ankle subtalar inversion internal moment was the most pronounced change. We speculate that the reduction in this moment is likely to reflect fatigue-related changes in muscles that resist subtalar inversion at the beginning of the stance phase during walking. For example, a previous study observed that the peroneal longus, i.e., a muscle involved in the modulation of subtalar inversion, experienced fatigue during extended marching on a treadmill, as measured from the EMG spectrum^68^. The same study also suggested that this fatigue-related change is associated with a lateral shift in the foot’s center of pressure, which likely increases the risk of ankle sprain injuries^68^. We also observed an increase in the peak knee flexion angle at the 5-km mark, which has been associated with a decrease in leg stiffness and an increase in the contact time with the ground^69^. Regarding parameters that did not achieve the study-specific relevant-change criteria, we observed an increasing trend for 14 out of 20 subjects without reaching the Bonferroni-corrected significance threshold for the stride length at the 5-km mark while walking with a load, where the increasing trend is in agreement with a previous study^17^. As subjects fatigued, they likely increased their stride length to facilitate gait stability^7,70^. This adjustment may lead to overstriding, which can be detrimental because it increases GRF, hip and knee muscle forces, and the overall risk of stress fracture compared to walking with the preferred stride length^7^.

Our study has limitations. First, because the a priori power calculation to determine the sample size of the study used a less stringent significance threshold (alpha = 0.05) than the statistical analysis with multiple comparisons and Bonferroni correction (alpha = 0.0012), our study was not adequately powered for statistical significance inference under the more stringent Bonferroni-corrected alpha threshold. Therefore, our findings on statistical significance should be interpreted within an exploratory context and complemented by the corresponding effect sizes. Second, our results are limited to women, which prevents us from generalizing the findings to men. Nevertheless, these results provide valuable insights into exertion-induced effects on gait biomechanics in women, who are more prone to musculoskeletal injuries, including tibial stress fracture, with injury rates two to four times higher than those in men^71–73^. Third, we did not measure the state of fatigue of the subjects. However, we collected their RPE at each kilometer, which indicated that subjects perceived a hard level of exertion after walking with a load for 5 km or running without a load for 5 km. In addition, we assumed that a distance of 5 km was sufficient to induce fatigue for both conditions, which may not be true for all subjects. However, we based the design of our study on a meta-analysis involving 25 different studies where fatigue was assumed to be present after running without a load for 1.7 km at 5.1 m/s (~ 11 min)^74^. Fourth, we performed the experiments in a controlled environment with a level treadmill, which is not fully representative of U.S. Army marching conditions. However, we made this choice in order to reduce the number of confounding factors during the experiment. Fifth, we did not use functional calibration trials to scale the generic musculoskeletal model. Instead, consistent with multiple studies as discussed by Lund et al.^75^, we used an optimization-based method that relied on a static calibration trial to estimate the body-segment lengths and joint centers and axes, which subsequently enabled tracking of marker trajectories during the walking and running trials. Sixth, we estimated foot length using plantar pressure measurements from each subject. This approach may have underestimated the foot length due to possible incomplete foot contact during toe-off. However, because of this possibility, we compared our plantar-based foot length estimates with values derived from widely used anthropometric scaling equations for body-segment lengths^76^ and observed an average difference of 4%, indicating the feasibility of using plantar pressure measurements to estimate foot length. Finally, we modeled the ankle and knee joints as revolute joints, even though these joints have multiple planes of motion and load carriage may induce changes in frontal-plane (abduction/adduction) and transverse-plane (internal/external rotation) knee kinematics^45^. However, we focused our analyses on the primary sagittal-plane motion (flexion-extension), for which revolute and higher-fidelity knee joint models have been shown to have comparable performance in both healthy individuals^77^ and those with knee arthroplasty^78^.

## Conclusion

In this exploratory study, we assessed the effects of exertion on the biomechanics of young, healthy women who walked with a load and ran without a load for 5 km. We found that the peak trunk flexion angle was the only parameter that increased with an effect size larger than or equal to 0.80 for both conditions. However, the peak hip extension internal moment increased, peak ankle dorsiflexion internal moment decreased, and peak ankle subtalar inversion internal moment decreased and had effect sizes smaller than 0.70 for one condition and larger than 0.80 for the other condition, suggesting that these parameters may change in tandem in both conditions. For running without a load, we observed the largest decrease in the peak ankle dorsiflexion internal moment, which also reached the Bonferroni-corrected significance threshold (*p* < 0.0012), and for walking with a load, we observed the largest decrease in the peak ankle subtalar inversion internal moment, which also reached the significance threshold. By identifying exertional-induced changes in gait-related parameters for two exercise conditions, while reducing between-subject variability by studying the same subjects under both conditions, our study offers insights into biomechanical parameters that should be further investigated for association with musculoskeletal injuries caused by exertion-induced muscle fatigue. In addition, the identification of parameters that are consistently affected by exertion will help devise countermeasures to reduce the risk of musculoskeletal injury.

## Supplementary Information

Below is the link to the electronic supplementary material.

Supplementary Material 1

## Data Availability

All data will be made available following a written request to the corresponding author, along with a summary of the planned research.
