# Supplementary material for: Effect of exertion on the biomechanics of walking with load carriage and running in young, healthy women
Source: Sci Rep. 2026 Jun 5;16:26163. doi: 10.1038/s41598-026-55814-0 (PMC13490393; doi:10.1038/s41598-026-55814-0)
Supplement: Supplementary file 1 [file 41598_2026_55814_MOESM1_ESM.docx]

Effect of exertion on the biomechanics of walking with load carriage and running in young, healthy women

Jose E. Rubio^1,2^, Fabricio Canales^1,2^, Manivannan Subramaniyan^1,2^, Md. Arifuzzaman Arif^1,2^, Ankur Padhye^3^, Stacey Meardon^3^, John Willson^3^, and Jaques Reifman^1*^

^1^Department of Defense Biotechnology High Performance Computing Software Applications Institute, Defense Health Agency Research & Development, Medical Research and Development Command, Fort Detrick, MD, United States

^2^The Henry M. Jackson Foundation for the Advancement of Military Medicine, Inc., Bethesda, MD, United States

^3^Department of Physical Therapy, East Carolina University, Greenville, NC, United States

*Correspondence:

Jaques Reifman, Ph.D.

Department of Defense Biotechnology High Performance Computing Software Applications Institute

Defense Health Agency Research & Development

Medical Research and Development Command

ATTN: FCMR-TT, 504 Scott Street

Fort Detrick, MD 21702-5012

E-mail: jaques.reifman.civ@health.mil

Phone: + 1 301 639 3637

Fax: +1 301 619 1983

**Supplementary Material**

Supplementary Table S1. Heart rate and rating of perceived exertion at 0 km and after walking 5 km with a load or running 5 km without a load

| **Subject** | **Rating of perceived exertion** | | | | **Heart rate (BPM)** | | | |
| --- | --- | --- | --- | --- | --- | --- | --- | --- |
|  | **Walking with a 22.7-kg load** | | **Running without a load** | | **Walking with a 22.7-kg load** | | **Running without a load** | |
|  | **0 km** | **5 km** | **0 km** | **5 km** | **0 km** | **5 km** | **0 km** | **5 km** |
| S1 | 12: Somewhat hard | 19: Extremely hard | 11: Light | 18: Extremely hard | 122 | 161 | 143 | 192 |
| S2 | 11: Light | 13: Somewhat hard | 12: Somewhat hard | 16: Very hard | 100 | 101 | 137 | 175 |
| S3 | 9: Very light | 15: Hard | 9: Very light | 20: Maximal exertion | 123 | 135 | 150 | 198 |
| S4 | 8: Very light | 14: Hard | 11: Light | 19: Extremely hard | 131 | 143 | 164 | - |
| S5 | 11: Light | 14: Hard | 14: Hard | 17: Very hard | 113 | 129 | 158 | 186 |
| S6 | 11: Light | 17: Very hard | 9: Very light | 14: Hard | 135 | 163 | 130 | 184 |
| S7 | 7: Extremely light | 18: Extremely hard | 7: Extremely light | 17: Very hard | 117 | 170 | 152 | 186 |
| S8 | 11: Light | 18: Extremely hard | 7: Extremely light | 10: Light | 108 | 123 | 151 | 172 |
| S9 | 8: Very light | 15: Hard | 7: Extremely light | 15: Hard | 121 | 160 | 125 | 188 |
| S10 | 11: Light | 14: Hard | 12: Somewhat hard | 13: Somewhat hard | 123 | 142 | 133 | 176 |
| S11 | 7: Extremely light | 17: Very hard | 8: Very light | 18: Extremely hard | 130 | 151 | 161 | 182 |
| S12 | 15: Hard | 19: Extremely hard | 12: Somewhat hard | 16: Very hard | 132 | 156 | 154 | 174 |
| S13 | 8: Very light | 15: Hard | 7: Extremely light | 14: Hard | 124 | 129 | 167 | 177 |
| S14 | 13: Somewhat hard | 17: Very hard | 12: Somewhat hard | 15: Somewhat hard | 103 | 108 | 135 | 166 |
| S15 | 7: Extremely light | 15: Hard | 8: Very light | 17: Very hard | 118 | 141 | 75 | 183 |
| S16 | 11: Light | 14: Hard | 10: Light | 12: Somewhat hard | 108 | 136 | 137 | 184 |
| S17 | 7: Extremely light | 17: Extremely hard | 6: No exertion | 16: Very hard | 127 | 148 | 138 | 177 |
| S18 | 13: Somewhat hard | 15: Hard | 9: Very light | 13: Somewhat hard | 111 | 131 | 145 | 178 |
| S19 | 10: Light | 18: Extremely hard | 7: Extremely light | 17: Very hard | 129 | 179 | 150 | 190 |
| S20 | 7: Extremely light | 10: Light | 8: Very light | 18: Extremely hard | 116 | 151 | 167 | 188 |
| **Mean (SD)** | 10 (2) | 16 (2) | 9 (2) | 16 (3) | 120 (10) | 143 (20) | 144 (20) | 182 (8) |
| **Effect size** | 1.00 | | 1.00 | | 1.00 | | 1.00 | |
| ***p*-value** | **<0.001** | | **<0.001** | | **<0.001** | | **<0.001** | |

The ratings of perceived exertion are based on the Borg scale. We calculated the effect size using the rank-biserial correlation *r* and the *p*-value using the Wilcoxon signed-rank test based on all 20 subjects. Positive effect size values indicate that the parameter increased after walking or running 5 km compared to 0 km (i.e., the baseline). Bold font indicates that the parameter increase or decrease was statistically significant (*p* < 0.05). The data are presented as mean [±1 standard deviation (SD)]. BPM: beats per minute.

Supplementary Table S2. Spatiotemporal and joint kinematic parameters at 0 km and after walking with a load or running without a load for 5 km

| **Biomechanical**  **parameter** | **Walking with a 22.7-kg load** | | **Running without a load** | |
| --- | --- | --- | --- | --- |
|  | **0 km** | **5 km** | **0 km** | **5 km** |
| **Normalized stride length** | 0.87 (0.04) | 0.88 (0.03) | 1.06 (0.09) | 1.05 (0.10) |
| **Peak joint angle (degree)** |  |  |  |  |
| **Hip** |  |  |  |  |
| Flexion | 35.29 (4.44) | 36.14 (4.42) | 42.52 (4.35) | 42.84 (5.82) |
| Extension | 19.21 (5.68) | 20.48 (5.84) | 10.33 (4.82) | 10.47 (5.09) |
| Abduction | 11.37 (2.26) | 11.64 (2.51) | 7.40 (2.28) | 8.59 (2.67) |
| Adduction | 11.16 (3.05) | 12.83 (3.48) | 11.06 (3.73) | 12.21 (3.32) |
| External rotation | 7.80 (3.09) | 8.78 (2.83) | 8.97 (3.54) | 9.87 (3.77) |
| Internal rotation | 3.57 (4.61) | 4.50 (5.02) | 1.92 (3.72) | 1.90 (3.19) |
| **Knee** |  |  |  |  |
| Flexion | 75.11 (2.59) | 76.69 (3.03) | 95.04 (7.87) | 96.30 (8.50) |
| Minimum flexion | 7.01 (3.53) | 6.41 (3.84) | 14.53 (4.28) | 16.25 (4.53) |
| **Ankle** |  |  |  |  |
| Plantarflexion | 13.19 (4.61) | 13.81 (5.11) | 20.88 (5.83) | 21.22 (6.97) |
| Dorsiflexion | 11.93 (3.06) | 12.51 (3.16) | 24.32 (3.35) | 25.15 (3.34) |
| **Trunk** |  |  |  |  |
| Flexion | 10.65 (3.87) | 13.87 (4.76) | 13.97 (4.48) | 16.22 (4.79) |
| Minimum flexion | 7.79 (4.11) | 10.56 (4.66) | 6.98 (4.71) | 9.38 (5.07) |
| **Initial joint angle (degree)** |  |  |  |  |
| **Hip** |  |  |  |  |
| Flexion | 31.27 (5.12) | 31.85 (5.17) | 30.61 (3.93) | 30.58 (5.04) |
| Abduction | 9.15 (3.21) | 9.46 (3.61) | - | - |
| Adduction | - | - | 1.91 (2.57) | 2.94 (2.62) |
| External rotation | 3.06 (5.03) | 2.94 (4.99) | 3.11 (5.22) | 2.77 (5.85) |
| **Knee** |  |  |  |  |
| Flexion | 11.80 (3.64) | 12.85 (4.09) | 15.59 (4.19) | 17.39 (4.63) |
| **Ankle** |  |  |  |  |
| Dorsiflexion | 2.42 (2.99) | 2.24 (2.73) | 1.94 (5.02) | 1.15 (3.09) |
| **Trunk** |  |  |  |  |
| Flexion | 10.04 (3.87) | 13.36 (4.70) | 8.35 (4.33) | 10.38 (5.04) |

The data are presented as mean (±1 standard deviation).

Supplementary Table S3. Ground reaction force, joint internal moment, and joint reaction force at 0 km and after walking with a load or running without a load for 5 km

| **Biomechanical**  **parameter** | **Walking with a 22.7-kg load** | | **Running without a load** | |
| --- | --- | --- | --- | --- |
|  | **0 km** | **5 km** | **0 km** | **5 km** |
| **Ground reaction force (BW)** | 1.73 (0.10) | 1.76 (0.10) | 2.31 (0.17) | 2.30 (0.15) |
| **Peak joint moment (N∙m/kg)** |  |  |  |  |
| **Hip** |  |  |  |  |
| Flexion | 0.68 (0.16) | 0.63 (0.18) | 0.75 (0.15) | 0.76 (0.16) |
| Extension | 1.54 (0.12) | 1.65 (0.14) | 1.44 (0.25) | 1.55 (0.23) |
| Abduction | 1.45 (0.19) | 1.47 (0.18) | 1.69 (0.28) | 1.66 (0.29) |
| Adduction | 0.22 (0.13) | 0.30 (0.18) | 0.06 (0.03) | 0.07 (0.04) |
| External rotation | 0.48 (0.10) | 0.54 (0.10) | 0.50 (0.14) | 0.51 (0.12) |
| Internal rotation | 0.09 (0.05) | 0.07 (0.04) | 0.09 (0.06) | 0.07 (0.04) |
| **Knee** |  |  |  |  |
| Flexion | 0.82 (0.18) | 0.87 (0.18) | 0.43 (0.07) | 0.46 (0.07) |
| Extension | 0.75 (0.25) | 0.81 (0.25) | 1.90 (0.31) | 1.82 (0.30) |
| **Ankle** |  |  |  |  |
| Plantarflexion | 2.02 (0.17) | 1.98 (0.14) | 2.22 (0.40) | 2.21 (0.36) |
| Dorsiflexion | 0.18 (0.05) | 0.16 (0.05) | 0.09 (0.05) | 0.04 (0.02) |
| Subtalar eversion | 0.46 (0.11) | 0.45 (0.10) | 0.40 (0.24) | 0.42 (0.23) |
| Subtalar inversion | 0.03 (0.02) | 0.01 (0.00) | 0.03 (0.04) | 0.02 (0.02) |
| **Peak joint reaction force (BW)** |  |  |  |  |
| Hip | 8.01 (0.81) | 8.32 (0.80) | 8.97 (1.14) | 9.10 (1.06) |
| Knee | 6.55 (0.89) | 6.69 (0.93) | 10.85 (1.79) | 10.75 (1.71) |
| Ankle | 8.15 (0.83) | 8.07 (0.75) | 10.91 (1.40) | 11.11 (1.13) |

The data are presented as mean (±1 standard deviation).

Supplementary Table S4. Joint and total positive and negative work at 0 km and after walking with a load or running without a load for 5 km

| **Biomechanical**  **parameter** | **Walking with a 22.7-kg load** | | **Running without a load** | |
| --- | --- | --- | --- | --- |
|  | **0 km** | **5 km** | **0 km** | **5 km** |
| **Joint energy generation (J/kg)** |  |  |  |  |
| Hip | 0.43 (0.07) | 0.47 (0.07) | 0.31 (0.09) | 0.35 (0.09) |
| Knee | 0.25 (0.07) | 0.29 (0.06) | 0.28 (0.07) | 0.27 (0.07) |
| Ankle | 0.35 (0.09) | 0.34 (0.07) | 0.76 (0.13) | 0.70 (0.12) |
| **Joint energy absorption (J/kg)** |  |  |  |  |
| Hip | 0.07 (0.04) | 0.07 (0.05) | 0.09 (0.04) | 0.07 (0.03) |
| Knee | 0.18 (0.05) | 0.17 (0.06) | 0.41 (0.10) | 0.37 (0.08) |
| Ankle | 0.10 (0.05) | 0.10 (0.04) | 0.26 (0.11) | 0.28 (0.07) |
| **Total energy generation (J/kg)** | 1.04 (0.10) | 1.10 (0.08) | 1.36 (0.18) | 1.33 (0.19) |
| **Total energy absorption (J/kg)** | 0.36 (0.07) | 0.36 (0.08) | 0.76 (0.14) | 0.72 (0.12) |

The data are presented as mean (±1 standard deviation).
